# Proton signal enhancement of three orders of magnitude in high-field liquid-state NMR

**DOI:** 10.1038/s41467-026-77297-3

**Published:** 2026-09-01

**Authors:** Kajum Safiullin, Dmitrii Zasukhin, Masoud Minaei, Karel Kouřil, Gerrit E. Janssen, Arno P. M. Kentgens, Benno Meier

**Affiliations:** 1 Institute for Biological Interfaces 4, Karlsruhe Institute of Technology, Eggenstein-Leopoldshafen, Germany; 2 Magnetic Resonance Research Center, Institute for Molecules and Materials, Radboud University, Nijmegen, Netherlands; 3 Institute of Physical Chemistry, Karlsruhe Institute of Technology, Karlsruhe, Germany

**Keywords:** NMR spectroscopy, Physical chemistry, Solution-state NMR

## Abstract

Dissolution-dynamic nuclear polarization (d-DNP) is a hyperpolarization technique that is used to enhance the sensitivity of liquid-state nuclear magnetic resonance (NMR) in biophysical and chemical applications. D-DNP is used widely for the sensitivity enhancement of hetero-nuclei such as ^13^C, but it is barely used for ^1^H hyperpolarization, despite the abundance of ^1^H in organic matter. Here we show that OX063, a radical that has so far been used only for the hyperpolarization of low-*γ* nuclei such as ^13^C, may be used also to hyperpolarize ^1^H. This hyperpolarization route is enabled by the use of a klystron amplifier that provides three orders of magnitude more power than conventionally used solid-state microwave sources. Following hyperpolarization, the sample is ejected from the polarizer and liquefied, yielding ^1^H signal enhancements of  ~ 1000 in high-field NMR. The use of OX063 limits not only *T*_1_ relaxation during sample transfer, but we also show that it preserves the liquid-state *T*_2_, and therefore offers superior sensitivity and resolution. The applicability of this method to polarize ^1^H of selected amino acids is demonstrated.

## Abstract (web summary)

Nuclear magnetic resonance often suffers from low sensitivity. Here the authors demonstrate that, by hyperpolarizing protons using the radical OX063, one may achieve signal enhancements of more than 1000 in high-field solution NMR.

## Introduction

Nuclear magnetic resonance (NMR) is a compelling probe of the structural and dynamic properties of matter. However, the sensitivity of NMR is usually low^1^. The NMR signal is proportional to the spin polarization, which is the population difference between spin states in an external magnetic field. In thermal equilibrium, the spin polarization of ^1^H nuclei at 9.4 T and 300 K is only 32 ppm, representing a tiny fraction of spins contributing to the NMR signal.

The sensitivity of NMR may be boosted using a wide range of hyperpolarization techniques^2^. Some of these techniques can be applied directly in the liquid state. For molecules that can be hydrogenated or bind reversibly to a suitable catalyst, the sensitivity of liquid-state NMR can be increased via parahydrogen-induced polarization (PHIP)^3,4^ and signal amplification by reversible exchange (SABRE)^5–7^. Aromatic amino acids and related molecules can be hyperpolarized using a photo-sensitizer and light irradiation, a technique known as photochemically induced dynamic nuclear polarization (photo-CIDNP)^8,9^. In the Overhauser effect, one transfers electron spin polarization to nuclear spins using microwave irradiation of the electron transition^10,11^. In viscous media, one may also achieve such a polarization transfer using microwave irradiation of an electron-nuclear transition, a resonance condition known as the solid effect^12^.

Dissolution-dynamic nuclear polarization (d-DNP) is an indirect hyperpolarization technique for liquid-state NMR with the potential to achieve near unity spin polarization^13–18^. In d-DNP, a molecule of interest is dissolved in a glass-forming solvent doped with a radical. Then the sample is frozen and subjected to microwave irradiation at cryogenic temperatures and a magnetic field of a few Tesla. Microwave irradiation transfers electron spin polarization (which is close to unity at these conditions) to nuclear spins near the radical, from where it is then distributed across the sample due to spin diffusion. Once the sample is polarized, it is dissolved and the resulting liquid is transferred to a liquid-state NMR apparatus.

While d-DNP is used widely for ^13^C- and ^15^N-enriched molecules as well as ^19^F-bearing molecules^16,17,19^, it is used rarely for ^1^H hyperpolarization^20–22^, likely because of fast *T*_1_ relaxation during the sample transfer^23^. Various approaches have been developed to overcome this problem. Most notably, UV-induced radicals, that quench immediately after the DNP step and the following heat up, were used as polarizing agents for water^24,25^, affording a liquid-state polarization as high as 80% with a ^1^H *T*_1_ > 30 s^26^. The spin order of hyperpolarized water can be used to implement 2D NMR on biomolecules, however, the polarization of target molecules has so far remained below 1%^27–33^. Likewise, in conventional DNP experiments as well as in so-called rapid-melt DNP, the observed liquid-state ^1^H polarization levels are typically well below 1%^23,34–36^. While the Hilty group did report ^1^H polarization levels of 3.6% and 6.4% for folic acid and 4’-hydroxyazobenzene-2-carboxylic acid (HABA)^21,37^, the >100-fold dilution of the sample and the concomitant loss in mass sensitivity^38^ limit the transferability of their technique to applications in areas such as metabolomics or the affinity determination of weak binders.

Recently, an alternative approach to d-DNP, named bullet-DNP was introduced by some of us^39,40^. In bullet-DNP, the hyperpolarized solid sample is transferred within ~70 ms, and dissolved only near the point of liquid-state NMR detection. This leads to a lower sample dilution and allows to perform experiments with very high overall mass and concentration sensitivity^41^. The short transfer times in bullet-DNP may be favorable also for ^1^H hyperpolarization, but one has to anticipate fast relaxation in the solid state. This applies in particular in the presence of nitroxide radicals, which exhibit an electron paramagnetic resonance (EPR) spectrum that is wide compared to the ^1^H Larmor frequency, and which are typically used for ^1^H hyperpolarization in d-DNP.

Nitroxide radicals are so far the only stable radicals used for sensitivity enhancement in ^1^H d-DNP and bullet-DNP^36^ because their broad EPR spectrum enables ^1^H hyperpolarization via triple-spin-flips (TSFs)^42^. TSFs are a generalization of the DNP mechanisms known as cross effect and thermal mixing^18,42,43^. In TSFs, an electron-electron flip-flop combines with a nuclear spin flip. By selectively saturating either the low- or the high-field part of the EPR spectrum, one can ensure that TSFs can only flip the nuclear spin up or down, resulting in either positive or negative signal enhancement. The advantage of using nitroxide radicals is that one needs less than 100 mW of microwave power to selectively saturate a part of the EPR spectrum. This microwave power can be supplied by economic solid-state microwave sources. However, the dependence on nitroxide radicals also implies that a relatively high radical concentration is required to have a sufficient number of electron-electron pairs to drive the TSFs. Furthermore, the TSFs may drive fast nuclear relaxation and hetero-nuclear thermal mixing^44,45^ at low fields when the electron spins are no longer fully polarized.

It is well known that ^1^H hyperpolarization may also be achieved with narrow-band trityl radicals, which are characterized by an EPR spectrum that is narrow compared to the ^1^H Larmor frequency. At low fields of the order of 1 T, commercial EPR instruments provide enough microwave power to drive this process^46^, and it is even possible to perform pulsed DNP using the NOVEL sequence^47^. At high field, ^1^H hyperpolarization has been implemented for magic angle spinning DNP using a gyrotron with an output power of 40 W^48^.

Here, we use the trityl radical OX063 and drive electron-nuclear double-spin-flips (DSFs) directly at high field, using high-power microwave irradiation supplied by an extended interaction klystron (EIK) amplifier with a peak power of 100 W. The underlying DNP mechanism, the solid effect^49,50^, is not active in the absence of microwave irradiation. Since the DNP mechanism requires only a single electron, the radical concentration can be reduced, and we show that radical concentrations as low as 5 mM are sufficient to achieve substantial nuclear spin polarization on the order of 1%. Since OX063 is a narrowband radical, triple-spin-flips involving ^1^H are no longer possible, further limiting relaxation. Following hyperpolarization, we conduct dissolution experiments with bullet-DNP with only 20-fold dilution, and find that OX063 offers superior liquid-state resolution, with no observable ^1^H line-broadening upon dissolution and a typical ^1^H resolution of 5 Hz or better, while nitroxide radicals in otherwise equivalent experiments cause ^1^H line-broadening of up to 20 Hz. The method is demonstrated for acetate, pyruvate and several amino acids.

## Results

### Microwave setup

**Fig. 1:**
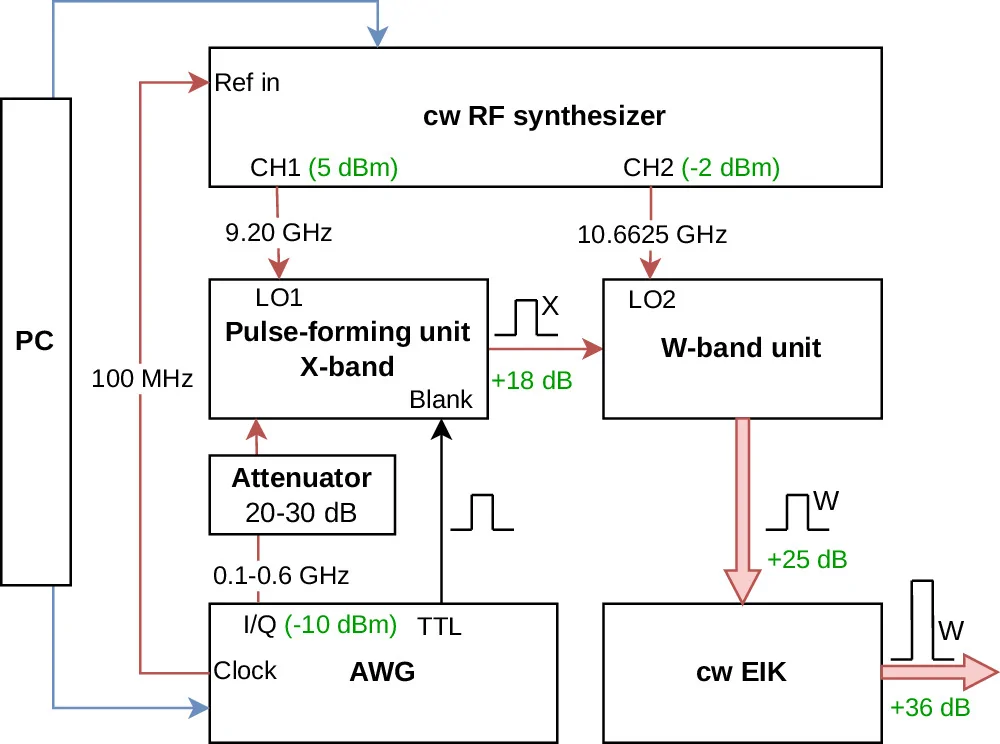
Pulsed high-power microwave setup used for bullet-DNP experiments. Radiofrequency pulses with desired durations are prepared as in-phase (I) and quadrature (Q) components at low frequency (0.1–0.6 GHz) using an arbitrary waveform generator (AWG). After attenuation (attenuation level affects the final output power), I/Q components are mixed in the X-band pulse-forming unit with a 9.2 GHz local oscillator signal (LO1) generated by a cw RF synthesizer. The pulse-forming unit amplifies RF pulses by 18 dB and is blanked according to TTL signals provided by the AWG. The resulting 9.3–9.8 GHz microwave pulses of desired duration enter the W-band unit through a 9.55 GHz bandpass filter (not shown). The microwave pulses are mixed with 8⋅LO2 (The 10.6625 GHz LO2 is also generated by the cw RF synthesizer) and the mixed signal is amplified by 25 dB by the W-band unit. The resulting 94.6–95.1 GHz pulses enter the continuous wave extended interaction klystron (cw EIK) through a WR-10 waveguide and are amplified by another 36 dB. The output of the EIK is connected to the DNP insert using several microwave waveguides. A computer (PC) is used to control the AWG and the cw RF synthesizer. Both latter units are synchronized with a 100 MHz clock.

The solid effect DNP mechanism requires high-power microwave to achieve a sufficient *B*_1_^51^. A block diagram of the high-power microwave setup used in this work is shown in Fig. 1.

A SynthHD.v2 (Windfreak Technologies, LLC, US) dual-channel continuous wave (cw) RF synthesizer is used to supply local oscillator (LO) signals at 9.2 GHz and 10.6625 GHz to a pulse forming unit and a W-band unit (both purchased from Bridge12, USA), respectively. A Keysight M8190A arbitrary waveform generator (AWG) additionally supplies I/Q data (0.2–0.6 GHz) to the I/Q mixer of the pulse-forming unit, which outputs pulses with phase and amplitude control at X-band. The W-band unit multiplies the incoming LO signal by 8, and then mixes the resulting signal with the X-band pulse. The W-band signal is then fed into a VKB2463 continuous wave extended interaction klystron amplifier (CPI, Canada) with 36 dB gain and a maximum output power of 97 W at 94.90 GHz at an RF bandwidth (−3 dB) of 300 MHz. Since the dynamic range of the AWG is limited, the output power is controlled additionally by variable RF attenuators at the input of the W-band extension unit.

The described microwave setup allows one to generate microwave pulses at desired duty cycles with durations from nanoseconds to milliseconds. The pulses are delivered from the klystron to the sample via a combination of a rectangular WR-10 waveguide, a rectangular to circular (4.8 mm) waveguide transition (Shanghai AT Microwave), a lab-built circular 4.8–9 mm waveguide transition, and a 9 mm inner diameter thin-wall steel tube.

### DNP spectrum

The specific feature of the solid effect is the presence of two antisymmetric peaks in the DNP profile that are separated by twice the nuclear Larmor frequency at the fixed external magnetic field. Hence, the solid effect occurs when *ω*_mw_ = *ω*_e_ ± *ω*_n_, where *ω*_mw_ is the microwave frequency, and *ω*_e_ and *ω*_n_ are the Larmor frequencies of the electron and the nuclear spin, respectively^12,50–52^

**Fig. 2:**
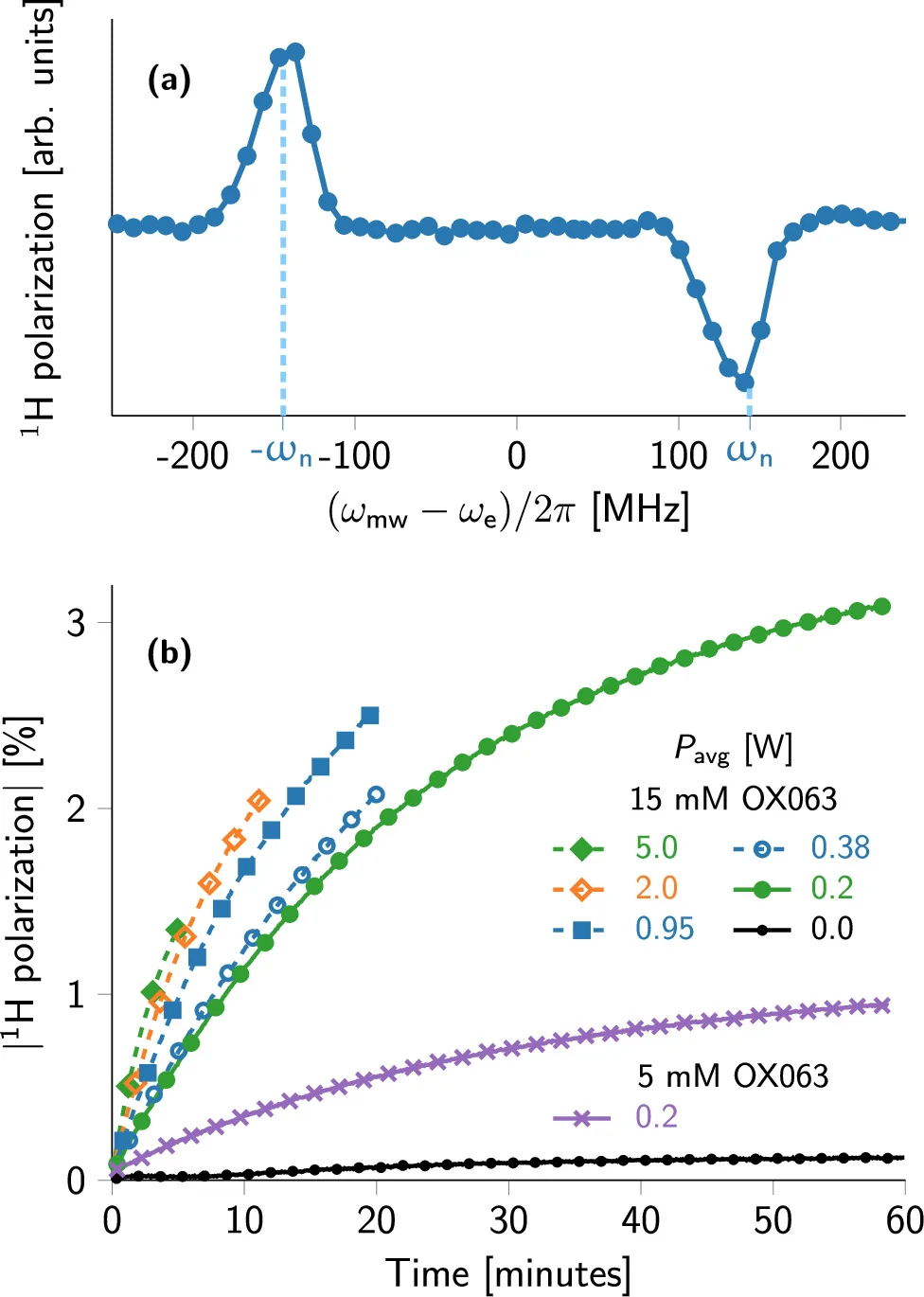
Solid-state ^1^H hyperpolarization with OX063. **a** Experimental ^1^H DNP spectrum of 0.5 M sodium acetate in DNP juice doped with 15 mM trityl OX063 radical at 1.5 K and 3.4 T. **b** ^1^H polarization build-up curves recorded at various microwave powers *P*_mw_ and duty cycles *D* at 3.4 T for 0.5 M sodium acetate in DNP juice doped with 5 mM and 15 mM trityl OX063 radical.

The DNP spectrum of 0.5 M sodium acetate in DNP juice doped with 15 mM OX063 (see Methods) is shown in Fig. 2a. A peak microwave power of 50 W was applied repeatedly for 9.6 μs, followed by no microwave irradiation for 86.4 μs, corresponding to a duty cycle of *D* = 0.1. The microwave frequency *ω*_mw_ was varied in 10 MHz increments around *ω*_e_ ≃ 94.9 GHz. For each increment, the ^1^H NMR signal intensity was recorded after 20 s of build-up time. Then the microwave was switched off for 25 s, allowing the sample to cool back down to the default 1.5 K temperature and a train of saturation NMR pulses was applied before the next polarization build-up started.

The resulting DNP spectrum, shown in Fig. 2a, is constructed from three DNP spectra that were recorded at slightly different external magnetic fields to compensate for the limited bandwidth of the EIK.

Clearly, there are two distinct peaks in the DNP spectrum, which are located at *ω*_e_ ± *ω*_n_. The spacing between their centers corresponds to 2*ω*_n_ with *ω*_n_ = 2*π* ⋅ 144.3 MHz, the Larmor frequency of ^1^H at 3.389 T, indicating the solid effect as the relevant DNP mechanism.

### DNP build-ups

Selected DNP build-up curves are shown in Fig. 2b. All curves were recorded using 2º-tipping angle NMR pulses at the negative lobe of the DNP spectrum, i.e. at the lower field peak of the DNP spectrum, which corresponds to a microwave frequency of 94.895 GHz at 3.383 T. Microwave pulses are applied continuously at *P*_mw_ = 20–95 W and duty cycle *D* = 4 ⋅ 10^−3^–10^−1^, but the average microwave power *P*_avg_ is shown for simplicity, please refer to the Supplementary Section I for details. The curves represent the polarization growth associated solely with DNP, since the background signal and thermally polarized signals are both subtracted (these are measured in a separate experiment without microwave irradiation).

The solid lines in Fig. 2 represent fits of the data to a mono-exponential growth function^53^:

1 $$M(t)={M}_{{{{{\rm{ss}}}}}}\left[1-\exp (-t/{T}_{{{{{\rm{DNP}}}}}})\right],$$

where *T*_DNP_ is the build-up time constant, and *M*_ss_ represents the resulting steady-state magnetization for *t* ≫ *T*_DNP_. The excellent agreement between data and fit, as well as the dependence of the build-up time constant on microwave settings indicate that the DNP process itself is the rate-limiting step, rather than the ensuing spin diffusion processes.

Both the increase of the microwave power and/or the pulsing duty cycle *D* lead to shorter polarization time constants *T*_DNP_ down to 4 minutes. A too high average microwave power *P*_avg_ > 0.5 W however leads to excessive heating of the sample and the surrounding helium bath, resulting in helium boil-off, and eventually a rapid increase of the sample temperature, leading to a premature termination of the buildup curve for an average microwave power of 0.4 W or more. The application of average microwave powers *P*_avg_ ≤ 0.2 W allowed us to polarize samples continuously for at least 1 h with the ability to start the new DNP experiment on the next sample within 15 min after sample transfer. This corresponds to ~75 min per sample in the routine-use operating regime.

The black curve in Fig. 2 is a buildup of the ^1^H signal without microwave irradiation, and is used to calculate the thermal equilibrium signal. This leads to an estimated steady-state polarization of ^1^H achieved in the solid state of up to −3.39% for hyperpolarization with *D* = 4 ⋅ 10^−3^, *P*_mw_ = 50 W (green curve in Fig. 2b, *T*_DNP_ = 25 min). These settings were used for the liquid-state experiments reported in Section “Liquid state”.

Since the solid effect is based on the hyperfine interaction between a nuclear spin and a single electron spin, it should still be effective at radical concentrations much lower than those used in experiments that rely on TSFs. Indeed, a 3-fold reduction in OX063 concentration from 15 to 5 mM leads to hyperpolarization with a resulting steady-state magnetization *M*_ss_ that is 3.5 times lower (−0.85 ± 0.07%) than that obtained for the sample with 15 mM OX063 (purple curve in Fig. 2b). For both radical concentrations the ^1^H polarization build-up time constant is approximately equal with *T*_DNP_ = 25 min.

### Liquid state

For liquid-state measurements, samples with OX063 concentrations of 5 and 15 mM were hyperpolarized for 1 h as described in the previous section. The samples were then ejected and shot into a 9.4 T magnet for dissolution and liquid-state NMR measurement. A set of free-induction-decays (FIDs) was recorded using 7.5º tipping angle pulses applied every second. This approach allows to assess the ^1^H polarization as well as the spin-lattice relaxation of the hyperpolarized ^1^H nuclei. The FID signal at thermal equilibrium was recorded after full equilibration using the same pulse but with 10 scans.

**Fig. 3:**
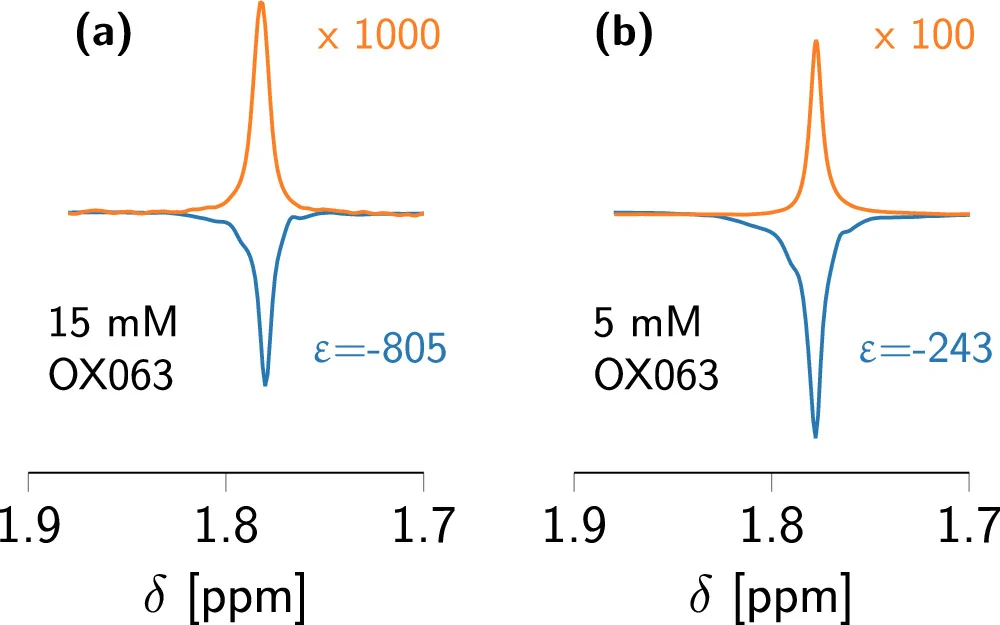
Hyperpolarized and thermal equilibrium spectra of CH_3_ group of acetate. The hyperpolarized ^1^H acetate NMR signal recorded immediately after sample dissolution and injection at 9.4 T is shown for **a** 15 mM and **b** 5 mM OX063 (blue). The corresponding thermal equilibrium signal (average of 10 scans) is shown for reference with readjusted amplitude as indicated (orange).

The methyl region of the ^1^H spectrum with the ^1^H acetate signal is shown for both samples in Fig. 3. The thermal equilibrium ^1^H signal is also shown for comparison. The liquid-state ^1^H NMR signal enhancement is *ε* = −805 at 9.4 T (polarization *P* = −2.6%) for the 15 mM trityl sample, and an enhancement of *ε* = −243 (*P* = −0.8%) is observed for the 5 mM trityl sample, in good agreement with the respective solid-state polarizations (−3.1% and −0.94%, respectively).

Bullet-DNP differs from conventional dissolution DNP by the level of sample dilution. While the low dilution of bullet-DNP preserves analyte concentration, it may lead to a higher concentration of radicals during the liquid-state NMR measurement. While the current state-of-the-art bullet-DNP apparatus achieves narrow NMR linewidths for ^13^C by pressurizing the sample^40,41,54^, it turns out that ^1^H experiments with broadband nitroxide radicals still exhibit significant linebroadening.

**Fig. 4:**
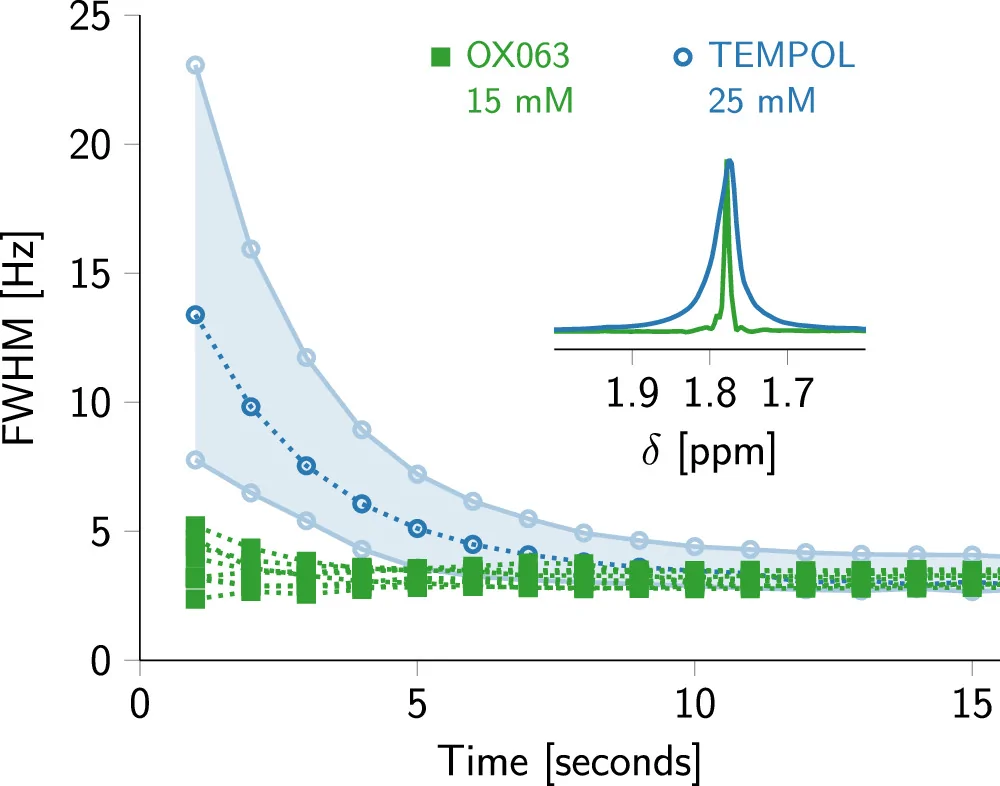
Evolution of the sodium acetate CH_3_ group ^1^H NMR linewidth (full width at half maximum, FWHM) over time after sample dissolution. Two selected sodium acetate spectra, acquired 1 s after dissolution, are shown on the inset. Note that the signals are labeled with the radical concentrations used during DNP for consistency. The nominal radical concentration (assuming a fully homogeneous distribution) in solution is 21-fold lower.

Acetate ^1^H spectra for samples with TEMPOL and OX063 are shown in the inset of Fig. 4. For consistency, the spectra are labeled with the radical concentrations that were used during hyperpolarization in the solid-state. After dilution and equilibration, the radical concentrations in solution are ~21 times lower. The sample polarized with OX063 clearly exhibits much higher resolution.

It turns out that this effect is highly repeatable. The main panel of Fig. 4 shows the linewidths from 6 independent dissolution experiments with OX063. The sodium acetate linewidths one second after the dissolution are in the range of 2.5–6 Hz (corresponding to a chemical shift resolution better than 0.01 ppm), with a small dependence on whether the positive or negative lobe of the DNP spectrum is used during the DNP build-up. Moreover, the linewidths equalize with the thermal equilibrium signal linewidth rapidly, within 1–2 s. It should be noted that in some experiments the observed line narrowing may be attributed to the RASER effect (Radiofrequency Amplification by Stimulated Emission of Radiation)^55,56^ during the first FID.

Linewidth data from TEMPOL with concentration of 25 mM (these values are typically used in ^1^H DNP^17^) were aggregated from 25 random ^1^H bullet-DNP experiments, see Supplementary Section II for details. Again, the concentrations refer to the radical concentration in the solid-state during DNP. In Fig. 4, we show, for simplicity, the lowest, the highest, and the mean FWHM values observed across all TEMPOL-based ^1^H experiments. In case of TEMPOL the linewidths are broadened significantly after dissolution (16 ± 8 Hz) and approach steady-state values over a timespan of 5–10 s.

Having established the hyperpolarization protocol, we performed a series of proof-of-concept experiments on small molecules including selected amino acids to demonstrate the applicability of the method to biologically relevant molecules. In these experiments we used 15 mM OX063 in the solid state and bullet-DNP conditions similar to the sodium acetate experiments (Fig. 3a) to hyperpolarize ^1^H of tryptophan, alanine, phenylalanine, sodium acetate and sodium pyruvate. The solvent composition was varied in order to demonstrate the efficiency of the suggested approach regardless of the solvent including a physiologically-friendly phosphate buffer solution with pH = 7.4 (PBS), which is frequently used in ligand-binding experiments.

**Fig. 5:**
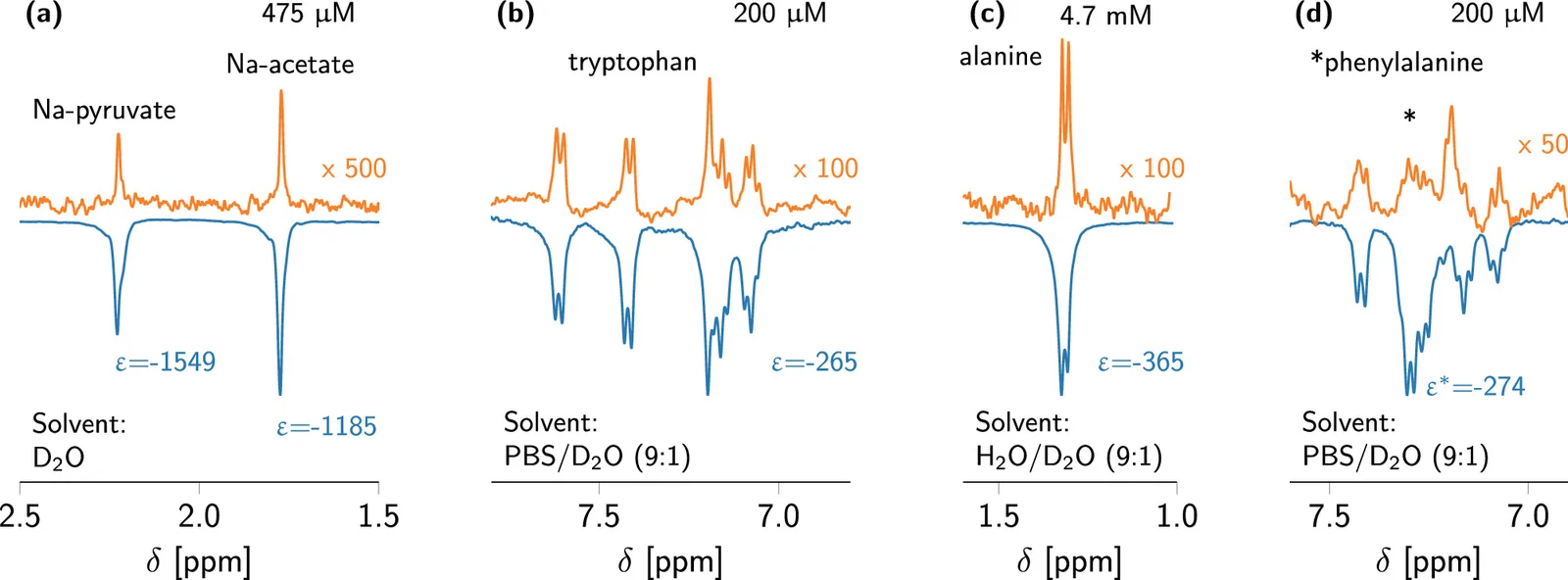
Hyperpolarized (blue) and corresponding thermal equilibrium (orange) ^1^H NMR spectra of various small molecules in liquid state at 9.4 T and room temperature obtained after bullet-DNP experiments with OX063 (15 mM in solid state) and subsequent dissolution in various solvents. **a** 475 μM sodium pyruvate and sodium acetate dissolved in D_2_O; **b** aromatic region of 200 μM tryptophan dissolved in a mixture of phosphate buffer: PBS/D_2_O (9:1); **c** 4.7 mM alanine dissolved in H_2_O/D_2_O (9:1); **d** a mixture of 200 μM phenylalanine (asterisk) and 200 μM tryptophan dissolved in a mixture of phosphate buffer: PBS/D_2_O (9:1). Hyperpolarized signals are recorded immediately after sample dissolution and injection. The corresponding thermal equilibrium signals are shown for reference with readjusted amplitudes as indicated, the averages of **a** 128, **b** 2048, **c** 10, and **d** 128 scans are shown; refer to Supplementary Section V for details. The concentrations are given for the liquid state, more details are given in Table 1.

The hyperpolarized and thermal equilibrium liquid-state NMR spectra obtained from the corresponding target molecules are shown in Fig. 5. The results are summarized in Table 1 and confirm high polarization levels in the liquid state, even when the ^1^H *T*_1_ is very short.

**Table 1:** ^1^H bullet-DNP results on various target molecules using OX063 radical

Target molecule	Concentration in		OX063 concentration	Solvent	*T*_1_ [s]	*ε*	*P* [%]
	Solid state	Liquid state					
Sodium acetate	0.5 M	23.8 mM	15 mM	H_2_O/D_2_O (9:1)	4.7 ± 0.1	−805	−2.6
Sodium acetate	0.5 M	23.8 mM	5 mM	H_2_O/D_2_O (9:1)	4.9 ± 0.1	−243	−0.8
Sodium acetate	10 mM	475 μM	15 mM	D_2_O	7.1 ± 0.1	−1185	−3.8
Sodium pyruvate	10 mM	475 μM	15 mM	D_2_O	8.2 ± 0.1	−1549	−5.1
Tryptophan	4.2 mM	200 μM	15 mM	PBS/D_2_O (9:1)	0.8 ± 0.2	−265	−0.85
Alanine	0.1 M	4.7 mM	15 mM	H_2_O/D_2_O (9:1)	1.2 ± 0.2	−365	−1.2
Phenylalanine	4.2 mM	200 μM	15 mM	PBS/D_2_O (9:1)	–	−274	−0.90

The quoted *T*_1_ values are obtained from the decay of the hyperpolarized signal, taking the effect of the RF pulses into account. This decay was measured for all molecules except for phenylalanine. In the case of tryptophan different protons contribute to the signal, such that the given value is an effective decay constant. The error analysis is detailed in the SI.

## Discussion

In this paper, we report ^1^H polarization levels for a range of molecules and a variety of aqueous solvents. The highest observed liquid-state ^1^H polarization is 5.1% for pyruvate in D_2_O, corresponding to a signal enhancement of 1550 at 9.4 Tesla. This corresponds to an increase by approximately an order of magnitude compared to previous work with conventional dissolution-DNP^57^. For the amino acids tryptophan, alanine and phenylalanine, polarizations of the order of 1% are obtained in almost fully protonated buffer. This polarization level is approximately an order of magnitude larger than the one previously observed using conventional dissolution-DNP with deuterated methanol^57^. Our approach therefore simultaneously boosts the sensitivity and it enables future applications such as the observation of ligand binding in near-physiological buffers.

While a higher polarization always increases sensitivity, our approach additionally benefits from a low 21-fold dilution, compared to the typically 100-fold dilution in conventional DNP. In general, sensitivity depends not only on polarization and concentration, but also on spectral resolution and the type of instrument used for signal detection. While a high-dilution may be perfectly acceptable for experiments in which a readily available target molecule needs to be observed at low concentrations, the same is not true for the observation of mass-limited samples that are best observed at the highest possible concentration. A comparison of our method with earlier d-DNP studies is given in the Supplementary Section IV. We refer the reader to the supplementary information of ref. ^41^ for a detailed analysis of the effect of liquid-state NMR probes, and to ref. ^38^ for a detailed analysis of sensitivity in hyperpolarized NMR.

With respect to the experiments reported here, there are several ways to further increase the ^1^H steady-state polarization in the solid state. One may use larger helium baths for sample cooling and apply stronger microwave irradiation. The observed difference between the polarization of 5 mM and 15 mM OX063 in the solid state suggests that a further increase in radical concentrations may increase the achieved polarization proportionally, although clustering may lead to diminishing returns at excessive radical concentrations^52,58^. Likewise, further deuteration may reduce ^1^H spin diffusion and relaxation, and increase the resulting ^1^H polarization.

The addition of small quantities of rare earth ion complexes shortens the trityl electron spin-lattice relaxation time^59^, which may improve the DNP process due to a shorter recovery time of the electron spin after a polarization transfer event. The benefits of a short electron *T*_1_ at low temperatures have to be weighed against potential polarization losses due to a faster nuclear *T*_1_ in the liquid state^60,61^.

Pulsed DNP techniques, such as chirp microwave pulses^62^, optimized versions of a chirp sequence, such as the adiabatic solid effect^63^, or optimized sequences and pulses such as time-optimized DNP^64^ or the X-inverse-X sequence^65^ may be used to drive the DNP process more efficiently, which may be advantageous due to the limited cooling capacity of the DNP system used in this work.

The narrow ^1^H linewidth that is observed in solution not only provides higher resolution, but also leads to a further sensitivity increase compared to the use of nitroxide radicals. While the origin of the observed, transient linebroadening in the presence of TEMPOL radicals requires further investigation, the high-resolution provided by trityl radicals directly benefits applications of the methodology presented here in metabolomics (which so far has been limited mostly to ^13^C hyperpolarization^66^). The recently proposed PEARLScreen probes ligand-binding with high sensitivity, and at very low protein concentrations^67^. Since our methodology preserves a long *T*_2_, it will be ideally suited to further reduce the required ligand and protein concentrations for this assay.

## Methods

### Sample preparation

**Fig. 6:**
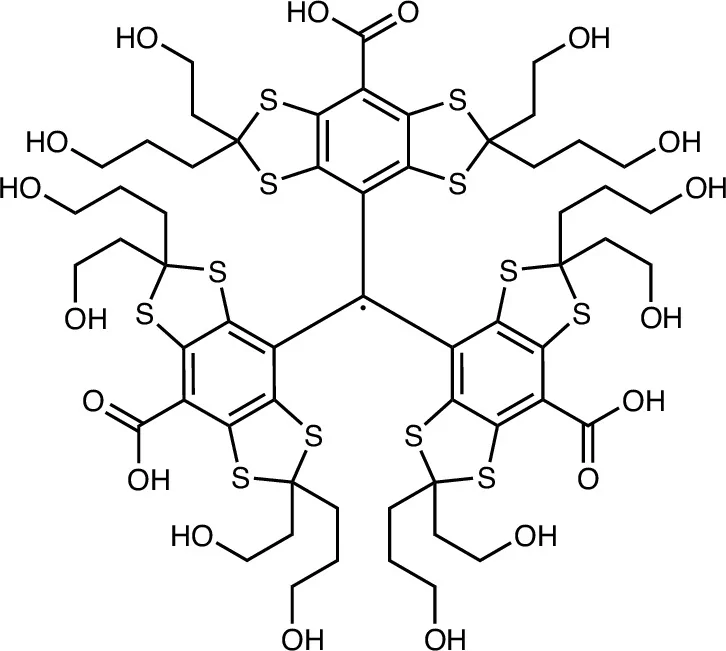
The molecular structure of OX063. This narrow-line OX063 radical was used in reported DNP experiments.

Experiments were carried out on samples containing 0.5 M ^13^C-labeled sodium acetate (1–^13^C–AcONa) dissolved in a mixture of H_2_O:D_2_O:*d*_8_–glycerol with a volume ratio 1:3:6 widely referred to as “DNP juice"^17^. The samples were doped with either 5 mM or 15 mM narrow-line radical trityl (OX063). The molecular structure of OX063 is shown in Fig. 6. After vortex mixing and at least 15 min of ultrasonication, a 40 μL aliquot of the samples was pipetted into an ultra-high molecular weight polyethylene (UHMW-PE) bullet for bullet-DNP experiments^41^. The sample was then frozen to liquid nitrogen temperature to form an amorphous glassy matrix as required for efficient DNP.

Experiments with broad-line radicals were performed on the same samples but doped with 25 mM of 4-hydroxy-2,2,6,6-tetramethylpiperidinyloxyl (TEMPOL) radical instead of trityl. In this case, glycerol of DNP juice was either protonated or deuterated, refer to Supplementary Information for details.

OX063 radical was obtained from GE HealthCare. All other chemicals were acquired from Sigma–Aldrich. The samples containing amino acids were prepared in a similar way and dissolved in a DNP juice with 15 mM OX063 radical. They contained, respectively, 4.2 mM L–tryptophan (Sigma–Aldrich); 0.1 M (S)–(+)–alanine (Merck KGaA); a mixture of L–tryptophan (Sigma–Aldrich), L–phenylalanine (Alfa Aesar, ThermoFisher) - 4.2 mM each; a mixture of L–tryptophan (Sigma–Aldrich), sodium pyruvate (Sigma–Aldrich), sodium acetate (Sigma–Aldrich) - 20 mM each.

### Solid-state (DNP) setup

The lab-built bullet-DNP insert that was used for DNP experiments, is equivalent to the one described by Kouřil et al.^40^. It was placed inside a Cryogenic Limited (UK) cryogen-free magnet with an integrated 50 mm variable temperature insert (VTI). The magnetic field was set to 3.4 T and slightly varied when necessary. The VTI temperature was set to 1.5 K and this temperature was used as the default sample temperature at the start of every DNP experiment. The temperature was monitored during microwave irradiation by a calibrated Cernox temperature sensor placed in the VTI space near the insert’s probehead. An Allen-Bradley carbon composition resistor placed near the sample container was used to confirm whether the sample was still immersed in a liquid helium bath.

In reported experiments, 9.6-μs-long microwave pulses were applied at duty cycles *D* between 4 ⋅ 10^−3^ and 1 ⋅ 10^−1^. The nominal power of microwave pulses *P* was varied between 20 and 95 W and frequencies *ω*_mw_ from 94.7 to 95.1 GHz range were used. That corresponds to a range of average EIK output power *P*_avg_ = *P* ⋅ *D* from 80 mW to 5 W.

A 95 GHz transmitter VDI-Tx-S159 (Virginia Diodes Inc., US) was used as a microwave source in additional experiments with TEMPOL radicals. In these experiments, 7 mW microwave irradiation was applied continuously at 94.88 GHz, the frequency that provides the maximum polarization at 3.389 T.

The insert was equipped with a ^1^H NMR detection circuit tuned to 144 MHz, the Larmor frequency of ^1^H at 3.4 T. A Bruker Avance Neo spectrometer was used to observe the ^1^H polarization build-up.

After the DNP process, the sample container was ejected and the sample was dissolved as described in ref. ^40^.

### Liquid-state setup

The sample dissolution and liquid state NMR experiments were performed at room temperature in the lab-built dissolution device similar to the one described by Kouřil et al.^40^ with the following parts changed. A solvent reservoir is now combined with a pinch valve within a same titanium piece. This shortens the distance between the NMR tube and the solvent reservoir and allows us to position the solvent reservoir closer to the center of the magnetic field. The dissolution of the sample therefore occurs at 8 T magnetic field instead of 1.3 T^40^. The dissolution device was placed inside a 9.4 T Bruker magnet and 800 μL of H_2_O:D_2_O (*v*:*v* = 9:1) mixture was used as a solvent for the sample. A 400 MHz Bruker Avance Neo spectrometer equipped with a TBO probe was used to observe ^1^H NMR.

The sample was injected into a 3-mm NMR tube within one second after dissolution and an FID was recorded every second. Then the NMR signal of the sample under thermal equilibrium conditions was recorded using the same settings with 10 scans to increase the signal to noise ratio.

For the scope-demonstration experiments the various solvents were used, as well as the NMR settings to record NMR signals under thermal equilibrium conditions (refer to Table 1 and Supplementary Section III for details).

The pynmr processing software^68^ was used to process and analyze the NMR data.

## Supplementary information


Supplementary Information
Transparent Peer Review file


## Data Availability

The raw data and processing that support the findings of this article, as well as the figure source data, have been deposited in KITopen^69^.

## Code availability

The processing codes that support the findings of this article have been deposited in KITopen^69^.
