# Supplementary material for: Proton signal enhancement of three orders of magnitude in high-field liquid-state NMR
Source: Nat Commun. 2026 Sep 1;17:9365. doi: 10.1038/s41467-026-77297-3 (PMC13534644; doi:10.1038/s41467-026-77297-3)
Supplement: Supplementary file 1 [file 41467_2026_77297_MOESM1_ESM.pdf]

SUPPLEMENTARY INFORMATION TO:

Proton signal enhancement of three orders of magnitude in high-field liquid-state NMR

Kajum Safiullin,[1,*] Dmitrii Zasukhin,[1] Masoud Minaei,[1] Karel Kouřil,[1]
Gerrit E. Janssen,[2] Arno P. M. Kentgens,[2] and Benno Meier[1,3,†]

[1]*Institute for Biological Interfaces 4, Karlsruhe Institute of Technology, Hermann-von-Helmholtz-Platz 1, Eggenstein-Leopoldshafen, 76344, Germany*
[2]*Magnetic Resonance Research Center, Institute for Molecules and Materials, Radboud University, Heyendaalseweg 135, Nijmegen, 6525AJ, Netherlands*
[3]*Institute of Physical Chemistry, Karlsruhe Institute of Technology, Fritz-Haber-Weg 2, Karlsruhe, 76131, Germany*

[*] kajum.safiullin@kit.edu
[†] benno.meier@kit.edu

I. DNP BUILD-UP PARAMETERS

Additional information on build-up curves plotted in Fig. 2b and corresponding DNP parameters, such as microwave pulse peak power P_{mw} and duty cycle D, are presented in Table I. The fit results according to the following equation are also specified in Table I:

$$M(t) = M_{\mathrm{ss}}\left[1 - \exp(-t/T_{\mathrm{DNP}})\right]. \tag{1}$$

Supplementary Table I. Information on build-up curves plotted in Fig. 2b. Build-up constant T_{DNP} time and M_{ss} are results of fits to Eq. (1), coefficients of determination R^2 are included. Uncertainties represent standard deviations.

OX063 concentration [mM]	P_{mw} [W]	D	P_{avg} [W]	T_{DNP} [minutes]	M_{ss} [%]	R^2
15	20	$1 \cdot 10^{-1}$	2.0	8.8±0.1	-2.77±0.01	0.99966
	50	$1 \cdot 10^{-1}$	5.0	3.9±0.1	-1.81±0.02	0.99943
	50	$4 \cdot 10^{-3}$	0.2	23.6±0.1	-3.39±0.01	0.99990
	95	$4 \cdot 10^{-3}$	0.38	24.2±0.2	-3.60±0.02	0.99978
	95	$1 \cdot 10^{-2}$	0.95	14.8±0.1	-3.32±0.01	0.99981
5	50	$4 \cdot 10^{-3}$	0.2	21.5±0.2	-1.04±0.01	0.99737

The coefficient of determination R^2 is calculated as follows:
For a dataset that contains n values y_i $(y_1, y_2, \ldots, y_n)$ and associated fit values f_i $(f_1, f_2, \ldots, f_n)$,

- the mean value:

$$\overline{y} = \frac{1}{n}\sum_i^n y_i; \tag{2}$$

- the residual sum of squares:

$$SS_{\mathrm{res}} = \sum_i^n (y_i - f_i)^2; \tag{3}$$

- the total sum of squares:

$$SS_{\mathrm{tot}} = \sum_i^n (y_i - \overline{y})^2; \tag{4}$$

- and the coefficient of determination:

$$R^2 = 1 - \frac{SS_{\mathrm{res}}}{SS_{\mathrm{tot}}}. \tag{5}$$

II. SODIUM ACETATE LINEWIDTHS

Figure 1 shows the data of Fig. 4 of the article before it was aggregated. The evolution of the sodium acetate ^{1}H NMR linewidth over time after sample dissolution at 9.4 T and 300 K in bullet-DNP experiments is plotted.

The experiments with 25 mM of 4-hydroxy-2,2,6,6-tetramethylpiperidinyloxyl (TEMPOL) radical were performed with protonated glycerol–h_8 (cross symbols) and deuterated glycerol–d_8 (square symbols) of DNP juice.

The experiments with 15 mM of OX063 radical were performed with deuterated glycerol–d_8 (circle symbols) of DNP juice. Each color represents data points obtained within a single experiment.

All given radical concentrations refer to the solid-state sample. After dilution and equilibration, the radical concentrations in solution are approximately 21 times lower.

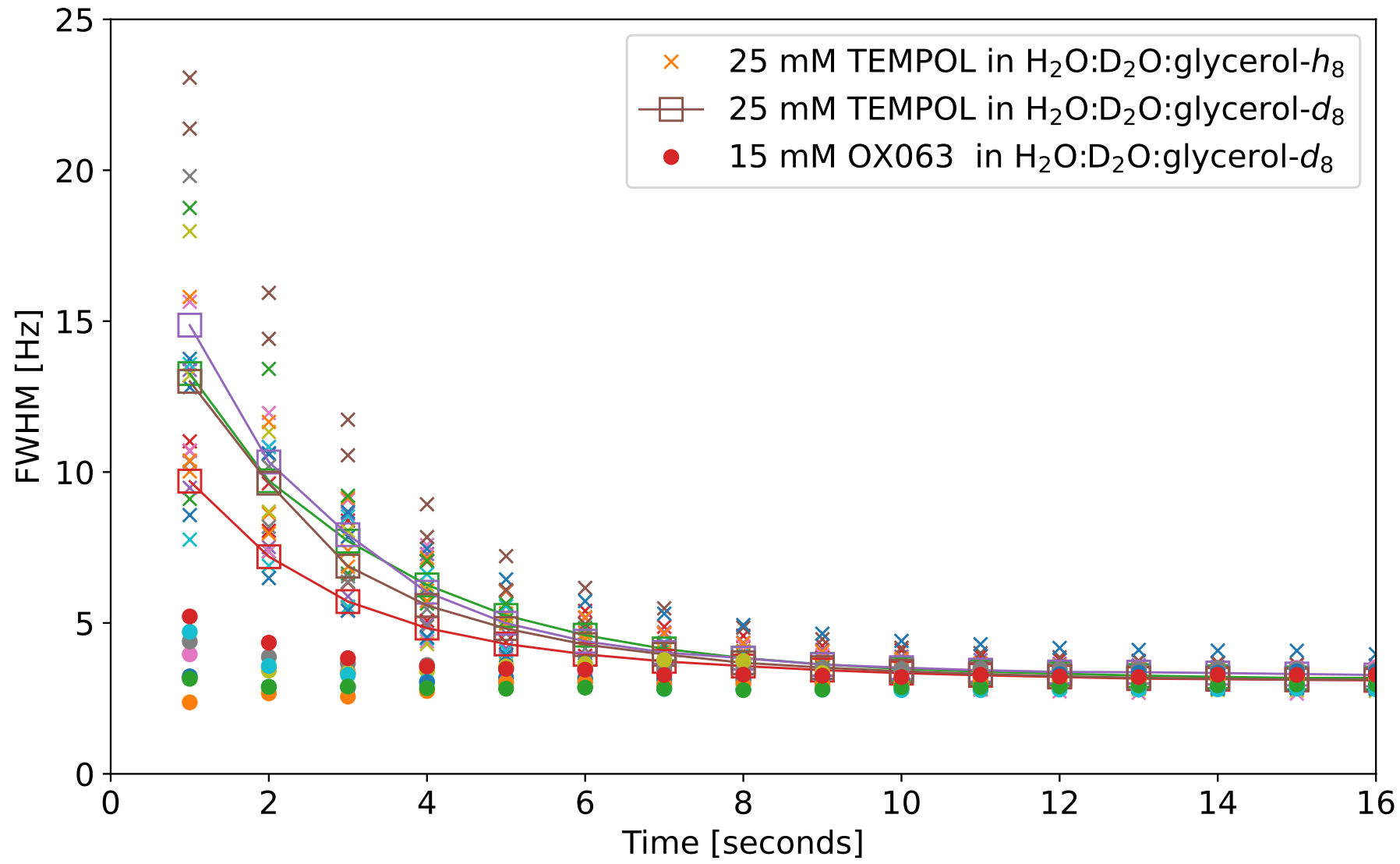

Supplementary Figure 1. Evolution of the sodium acetate ^{1}H NMR linewidth over time after sample dissolution.

III. DEMONSTRATION NMR EXPERIMENTS SETTINGS

Supplementary Table II. NMR settings used to acquire both polarized and thermal ^{1}H NMR FID signals for various target molecules, solvents, tipping angles pulses, number of scans NS, and receiver gain RG

<table>
<tr><th rowspan="2">index</th><th rowspan="2">target molecules</th><th rowspan="2">solvent</th><th rowspan="2">tipping angle [°]</th><th colspan="2">Hyperpolarized</th><th colspan="2">Thermal equilibrium</th></tr>
<tr><th>NS</th><th>RG</th><th>NS</th><th>RG</th></tr>
<tr><td>1</td><td>sodium pyruvate/ sodium acetate</td><td>D_2O</td><td>7</td><td rowspan="4">1</td><td rowspan="4">1</td><td>128</td><td rowspan="3">1</td></tr>
<tr><td>2</td><td>tryptophan</td><td>PBS (pH=7.4)/D_2O (9:1)</td><td>90</td><td>2048</td></tr>
<tr><td>3</td><td>alanine</td><td>H_2O/D_2O (9:1)</td><td>7</td><td>10</td></tr>
<tr><td>4</td><td>tryptophan/ phenylalanine</td><td>PBS (pH=7.4)/D_2O (9:1)</td><td>90</td><td>128</td><td>32</td></tr>
</table>

IV. DILUTION FACTOR AND OBSERVED SAMPLE VOLUME

Let us consider how dilution affects the observed NMR signal. The hyperpolarized frozen solid a volume V_{solid}.

In both D-DNP and bullet-DNP, this solid is then dissolved using an amount of solvent V_{solvent}. The amount of solvent used is greater than the volume of the NMR coil used for detection of the liquid-state signal, such that only a fraction of the hyperpolarized analyte ends up in the NMR tube. In D-DNP, the volume is typically of the order of several mL in order to provide sufficient heat to dissolve the solid and flush the liquid out of the cryogenic sample environment. In bullet-DNP, the volume is chosen such that the NMR tube can be filled reliably and completely in order to attain a good shim. Assuming a homogeneous concentration of the analyte, this yield is

$$f = \frac{V_{\mathrm{NMR}}}{V_{\mathrm{solvent}} + V_{\mathrm{solid}}} \tag{6}$$

For applications that require a high mass sensitivity, such as metabolomics, one seeks to have $f \sim 1$. (Note that the concentration of the analyte is not necessarily very homogeneous in conventional D-DNP experiments [1]). The implication of $f \ll 1$ is that most of the hyperpolarized material does not end up in the NMR tube, and so does not contribute to the detected signal.

A comparison of yields for several studies is given in Tab. III. In the current work, we use a 3 mm tube assembly (TgK Scientifc, UK), leading to a liquid volume inside the NMR coil of 95 µL. In the other cited works, 5 mm probes and 5 mm NMR tubes were used. For these studies, we assume regular NMR tubes with a wall thickness of 0.4 mm and a coil height of 2.1 cm, leading to an active volume of 300 µL.

Supplementary Table III. Dilution and yield in selected studies

source	V_{solid} [µL]	V_{solvent} [µL]	V_{NMR} [µL]	f
current work	40	800	95	0.113
Dey et al. [2]	200	5000	300	0.058
Wang et al. [3]	10	4000	300	0.075
Wang et al. [4]	30	4000	300	0.074

V. A COMPARISON OF OBTAINED POLARIZATIONS AND YIELDS FOR SEVERAL STUDIES

A comparison of polarizations and yields for several studies on the same target molecules in aqueous solutions is given in Tab. III. In the current work, we use a 3 mm tube assembly (TgK Scientifc Limited, UK), leading to a liquid volume inside the NMR coil of 95 µL. In the other cited works, 5 mm probes and 5 mm NMR tubes were used, except [5] (3 mm tube). For these studies, we assume regular NMR tubes with a wall thickness of 0.4 mm and a coil height of 2.1 cm, leading to an active volume of 300 µL.

Supplementary Table IV. Comparison of current work with other polarization methods applied to the same target molecules in aqueous solutions. Results with deuterated methanol, a toxic solvent for biological molecules, are shown for completeness since they yielded higher polarizations in one of the cited studies.

target molecule	source	method	solvent	P [%]	f
sodium acetate	current work	bullet-DNP	D_2O	-3.8	0.113
	current work	bullet-DNP	H_2O/D_2O (9:1)	-2.6	0.113
	Dey et al. [2]	d-DNP	D_2O	0.4	0.058
	Dey et al. [2]	d-DNP	CD_3OD	4.8	0.058
sodium pyruvate	current work	bullet-DNP	D_2O	-5.1	0.113
	Dey et al. [2]	d-DNP	D_2O	0.5	0.058
	Dey et al. [2]	d-DNP	CD_3OD	5.9	0.058
alanine	current work	bullet-DNP	H_2O/D_2O (9:1)	-1.2	0.113
	Dey et al. [2]	d-DNP	CD_3OD	0.13	0.058
	Zachrdla et al. [6]	Overhauser effect and HyperW	D_2O	0.02	∼0.5
	Aleshin et al. [7]	Overhauser effect and HyperW	D_2O	0.01	0.53
tryptophan	current work	bullet-DNP	PBS/D_2O (9:1)	-0.85	0.113
	Torres et al. [5]	photo-CIDNP	PBS	0.36	∼0.35
phenylalanine	current work	bullet-DNP	PBS/D_2O (9:1)	-0.90	0.113

SUPPLEMENTARY REFERENCES

[1] Elliott, S. J. *et al.* Practical dissolution dynamic nuclear polarization. *Prog. Nucl. Magn. Reson. Spectrosc.* **126-127**, 59–100 (2021).

[2] Dey, A., Charrier, B., Ribay, V., Dumez, J.-N. & Giraudeau, P. Hyperpolarized ^{1}H and ^{13}C NMR spectroscopy in a single experiment for metabolomics. *Anal. Chem.* **95**, 16861–16867 (2023).

[3] Wang, Y. & Hilty, C. Amplification of nuclear Overhauser effect signals by hyperpolarization for screening of ligand binding to immobilized target proteins. *Anal. Chem.* **92**, 13718–13723 (2020).

[4] Wang, Y., Kim, J. & Hilty, C. Determination of protein–ligand binding modes using fast multi-dimensional NMR with hyperpolarization. *Chem. Sci.* **11**, 5935–5943 (2020).

[5] Torres, F. *et al.* Molecular features toward high photo-CIDNP hyperpolariztion explored through the oxidocyclization of tryptophan. *Phys. Chem. Chem. Phys.* **23**, 6641–6650 (2021).

[6] Zachrdla, M. *et al.* Hyperpolarized nuclear Overhauser enhancement of alanine methyl groups by doubly relayed proton exchange. *J. Magn. Reson.* **364**, 107727 (2024).

[7] Aleshin, D., Butbul, K., Hahamy, I., Abergel, D. & Frydman, L. On the effects of hyperpolarized water-based dissolution on the solute and solvent ^{1}H NMR spectra of small molecules. *Phys. Chem. Chem. Phys.* (2026).
