# Supplementary material for: Proton signal enhancement of three orders of magnitude in high-field liquid-state NMR
Source: Nat Commun. 2026 Sep 1;17:9365. doi: 10.1038/s41467-026-77297-3 (PMC13534644; doi:10.1038/s41467-026-77297-3)
Supplement: Supplementary file 2 [file 41467_2026_77297_MOESM2_ESM.pdf]

nature portfolio

Peer Review File

Proton signal enhancement of three orders of magnitude in high-field liquid-state NMR

Corresponding Author: Dr Kajum Safiullin

This file contains all reviewer reports in order by version, followed by all author rebuttals in order by version.

Version 0:

Reviewer comments:

Reviewer #1

(Remarks to the Author)
This is a technically strong and carefully executed manuscript. The combination of OX063 with high-power microwave irradiation to obtain liquid-state 1H enhancement near 1000, together with the linewidth comparison against TEMPOL, is an impressive experimental achievement. However, I am not yet convinced that the manuscript, in its current form, demonstrates a sufficiently clear advance in general utility, and several central claims would benefit from clarification and stronger quantitative support.

The main message and practical advance are not yet articulated clearly enough. As written, the principal result seems to be that, with very high microwave power, OX063 can indeed be used for high-field 1H hyperpolarization. This is a great technical achievement, but the manuscript does not yet make clear what this now enables in practice beyond showing that it can be done with acetate. Please explain more explicitly why this is a broadly important advance rather than primarily a specialist instrumental development.

The comparison to prior work based on >100-fold dilution is not clear enough. The manuscript currently mixes enhancement, concentration sensitivity, and mass sensitivity without providing a transparent apples-to-apples comparison. Please define exactly what metric is being compared and provide a quantitative comparison to the prior reports. As written, it is difficult to assess the claim that the present approach provides better practical sensitivity.

The statement that 5 mM OX063 yields “substantial polarization” is too vague. Please report explicit numerical polarization values (ideally in both solid and dissolved liquid state) for 5 mM and 15 mM samples.

From the reported liquid-state enhancement of about -200, the 5 mM case appears to correspond to only about 0.6% 1H polarization at 9.4 T; if that is correct, it should be stated explicitly. More generally, please reconcile the discussion around the 5 mM case in Fig. 2 with the substantially lower liquid-state enhancement reported in Fig. 3, as this part of the manuscript is currently confusing.

The manuscript emphasizes the availability of three orders of magnitude more microwave power, but the average power appears to remain strongly limited by heating and helium boil-off. This is important for judging the practical usefulness of the method. Please clarify whether the 25 s microwave-off period reflects a general operating constraint, and discuss more explicitly what the realistic throughput and routine-use operating regime would be.

The broader applicability claims should be either strengthened or toned down. The narrower liquid-state linewidths with OX063 relative to TEMPOL are important, but the evidence is still limited to a simple acetate model system. Statements about likely impact in metabolomics, weak-binder screening, or wider community adoption remain somewhat speculative without a more application-oriented demonstration.

The figure presentation can be improved. In Fig. 3, the vector graphic rendering should be corrected; the stroke connections produce noticeable sharp edges.

Reviewer #2

(Remarks to the Author)
The manuscript by Safiullin at al describes an experiment that hyperpolarizes the spin states of the protons in the methyl group of sodium acetate. This results in an enhancement of the NMR signal of about -800, corresponding to a spin polarization of ~ -2.6 %. The method is based on bullet dissolution dynamic nuclear polarization, which polarizes a sample containing a paramagnetic radical at liquid helium temperatures. The radical in the sample is saturated by quasi-continuous microwave irradiation at or close to the electron spin resonance. The sample is encapsulated in a bullet, which is rapidly transferred from the polarizer magnet into the NMR magnet. The NMR signal is subsequently detected in the liquid state after rapid dissolution of the sample. The bullet DNP method, developed by some of the authors, is a variant of the widespread dissolution d-DNP method. Goal of this paper is to overcome some general challenges related to sensitivity and resolution of d-DNP. One issue is a fast T1 relaxation of the hyperpolarized nuclei during the transfer between the two magnets, which is particularly severe for protons. A second important issue is the NMR resolution in the liquid state, which is aggravated by the presence of a paramagnetic radical.
Two interesting aspects are introduced in this work as compared to literature: 1) the use of a narrow line radical, a trityl radical, which allows to perform the DNP polarization step with the so-called solid effect mechanism. Because of the long relaxation times of this radical, polarization is better preserved and NMR lines are less affected by the paramagnetic radical; 2) the introduction of a pulsed high power microwave source, an extended interaction klystron multiplier (EIK) for pumping with peak power up to 100 Watt. The reported experiments are well performed. The authors demonstrate that the DNP mechanism is a solid-state effect and the enhancements depend on the duty cycle (or average power) from the source. They also show a narrowing of the NMR line as compared to the widespread nitroxide radical.

My main criticism concerns the overall claim and the way the manuscript is written in some parts. The method is demonstrated on a single molecule, particularly on a methyl group that is a special case due to its fast motion/rotation. In my opinion, this is too limited to claim that the method is suited to hyperpolarize protons on small molecules, with perspective applications in metabolomics and ligand binding studies. The manuscript would strongly benefit from a few more examples on small molecules.

It should be considered that there exists a whelm of literature on d-DNP using water protons and how this can be used to polarize proteins. The citations here could be expanded (see recent papers by Frydman, Bodenhausen, Kurzbach, etc). Also, citations on state-of-the art liquid-state NMR hyperpolarization, including Overhauser and solid-effect DNP as well as PHIP in liquids, are missed.

Moreover, the manuscript contains statements which are not well-explained. Examples are listed below. In my view, the manuscript is not suited to Nature Communications in the present form. It might become convincing if more examples and detailed comparison with current state-of-the art in liquid-state hyperpolarization methods are added.

Specific points:
1) Introduction: 'A broadly applicable technique for liquid-state NMR is d-DNP…' I'm not sure if this statement is correct and the technique is general. There is still no general method for liquids. The authors should cite here also other approaches like solid effect in liquids (Ref. 17 is cited at the wrong place), recent progress in Overhauser DNP at high fields as well as PHIP (see above).
2) Introduction, page 2: why only thermal mixing is discussed and not cross-effect with biradicals ? Please explain the difference for the general reader.
3) C: DNP build-ups. 'The curves represent the polarization growth associated with DNP as background..' This statement is unclear: what is the DNP as background ?
4) Same chapter: 'A threefold reduction in OX063 concentration…leads to a steady-state magnetization that is 3.5 larger than that obtained with 15 mM..'. This sentence seems wrong. From Figure 2, I read the opposite, ie the polarization of the 15 mM sample (green curve) is three time larger the one of the 5 mM sample (purple). Please clarify.
5) Discussion, first paragraph. Please give numbers for concentration sensitivity instead of dilution factors. The latter cannot be easily translated in sensitivity.
6) Discussion, third paragraph. Why does a shorter T1 of the trityl radical improves the DNP process ? Why do rare earth ions only affect electron T1 and not nuclear T1 ? Please expand on this.
7) Discussion, last paragraph: 'the high resolution provided by nitroxide radicals directly benefits applications of the methodology presented here in metabolomics..' The high resolution should refer to the OX063 radical instead of nitroxide. Moreover, the high resolution is demonstrated only on a methyl group, which is a special case as fast rotating. This statement is speculative.
8) Please expand the discussion and comparison to other methods for hyperpolarization in liquids.

Reviewer #3

(Remarks to the Author)
The article uses a version of dissolution DNP to polarize samples via 1H NMR rather than 13C NMR. The article is very well written and the experiments are complete and well documented. The rationale for using 1H rather than 13C is sensitivity and the shorter polarization periods which are well known. In this sense the paper is not breaking new ground. However, with some rare exceptions the dDNP community has be religiously focuses on proceeding with 13C polarization. The "bullet" polarizers developed by Meier facilitate using 1H.

I would favor publishing the article without modifications as it does demonstrate the way that dDNP experiments should be performed.

Version 1:

Reviewer comments:

Reviewer #1

(Remarks to the Author)
The authors have improved the manuscript, addressed the questions and expanded the scope of substrates. I will be happy to see their work published in Nature Communications.

Open Access This Peer Review File is licensed under a Creative Commons Attribution 4.0 International License, which permits use, sharing, adaptation, distribution and reproduction in any medium or format, as long as you give appropriate credit to the original author(s) and the source, provide a link to the Creative Commons license, and indicate if changes were made.
In cases where reviewers are anonymous, credit should be given to 'Anonymous Referee' and the source.
The images or other third party material in this Peer Review File are included in the article's Creative Commons license, unless indicated otherwise in a credit line to the material. If material is not included in the article's Creative Commons license and your intended use is not permitted by statutory regulation or exceeds the permitted use, you will need to obtain permission directly from the copyright holder.
To view a copy of this license, visit https://creativecommons.org/licenses/by/4.0/

We thank the reviewers for their comments and questions. Our responses are presented below in blue.

Reviewer #1 (Remarks to the Author):

This is a technically strong and carefully executed manuscript. The combination of OX063 with high-power microwave irradiation to obtain liquid-state 1H enhancement near 1000, together with the linewidth comparison against TEMPOL, is an impressive experimental achievement. However, I am not yet convinced that the manuscript, in its current form, demonstrates a sufficiently clear advance in general utility, and several central claims would benefit from clarification and stronger quantitative support.

The main message and practical advance are not yet articulated clearly enough. As written, the principal result seems to be that, with very high microwave power, OX063 can indeed be used for high-field 1H hyperpolarization. This is a great technical achievement, but the manuscript does not yet make clear what this now enables in practice beyond showing that it can be done with acetate. Please explain more explicitly why this is a broadly important advance rather than primarily a specialist instrumental development.

We have run additional experiments in which our methodology is applied not only to acetate, but also to pyruvate and several amino acids. We observe substantial enhancements even on rapidly decaying signals of aromatic protons, with an order-of-magnitude increase in polarization with respect to conventional dissolution-DNP. The new experiments are presented in detail in the revised manuscript as well as in the supplementary information.

The comparison to prior work based on >100-fold dilution is not clear enough. The manuscript currently mixes enhancement, concentration sensitivity, and mass sensitivity without providing a transparent apples-to-apples comparison. Please define exactly what metric is being compared and provide a quantitative comparison to the prior reports. As written, it is difficult to assess the claim that the present approach provides better practical sensitivity.

We now contrast our work in greater detail to a previous study that used conventional dissolution-DNP. However, sensitivity depends not only on polarization and concentration, but on a range of other parameters. We have added a paragraph to the discussion that addresses this issue and links to some of our previous work in which the sensitivity issue is discussed in detail.

The statement that 5 mM OX063 yields “substantial polarization” is too vague. Please report explicit numerical polarization values (ideally in both solid and dissolved liquid state) for 5 mM and 15 mM samples.

We now give explicit polarization values for all experiments in Table 1.

From the reported liquid-state enhancement of about -200, the 5 mM case appears to correspond to only about 0.6% 1H polarization at 9.4 T; if that is correct, it should be stated explicitly. More generally, please reconcile the discussion around the 5 mM case in Fig. 2 with the substantially lower liquid-state enhancement reported in Fig. 3, as this part of the manuscript is currently confusing.

That is correct. The value is now stated explicitly in Table 1, and the text has been rewritten for clarity.

The manuscript emphasizes the availability of three orders of magnitude more microwave power, but the average power appears to remain strongly limited by heating and helium boil-off. This is important for judging the practical usefulness of the method. Please clarify whether the 25 s microwave-off period reflects a general operating constraint, and discuss more explicitly what the realistic throughput and routine-use operating regime would be.

We have updated the manuscript. In all liquid-state hyperpolarization experiments, the microwave was applied with a given duty cycle for 1 h at optimal conditions. The 25 s delay is used only for measuring the DNP spectrum and appears only under the respective heading. It is correct that the duty cycle is low, and hence the average power is much lower than the peak power. We have added a comment regarding a possible routine use operation.

The broader applicability claims should be either strengthened or toned down. The narrower liquid-state linewidths with OX063 relative to TEMPOL are important, but the evidence is still limited to a simple acetate model system. Statements about likely impact in metabolomics, weak-binder screening, or wider community adoption remain somewhat speculative without a more application-oriented demonstration.

As detailed above we have expanded the scope of the manuscript and demonstrate hyperpolarization of other molecules, including amino acids, in a range of physiologically relevant solvents.

The figure presentation can be improved. In Fig. 3, the vector graphic rendering should be corrected; the stroke connections produce noticeable sharp edges.

Figure 3 has been revised accordingly.

Reviewer #2 (Remarks to the Author):

The manuscript by Safiullin at al describes an experiment that hyperpolarizes the spin states of the protons in the methyl group of sodium acetate. This results in an enhancement of the NMR signal of about -800, corresponding to a spin polarization of ~ -2.6 %. The method is based on bullet dissolution dynamic nuclear polarization, which polarizes a sample containing a paramagnetic radical at liquid helium temperatures. The radical in the sample is saturated by quasi-continuous microwave irradiation at or close to the electron spin resonance. The sample is encapsulated in a bullet, which is rapidly transferred from the polarizer magnet into the NMR magnet. The NMR signal is subsequently detected in the liquid state after rapid dissolution of the sample. The bullet DNP method, developed by some of the authors, is a variant of the widespread dissolution d-DNP method. Goal of this paper is to overcome some general challenges related to sensitivity and resolution of d-DNP. One issue is a fast T1 relaxation of the hyperpolarized nuclei during the transfer between the two magnets, which is particularly severe for protons. A second important issue is the NMR resolution in the liquid state, which is aggravated by the presence of a paramagnetic radical.
Two interesting aspects are introduced in this work as compared to literature: 1) the use of a narrow line radical, a trityl radical, which allows to perform the DNP polarization step with the so-called solid effect mechanism. Because of the long relaxation times of this radical, polarization is better preserved and NMR lines are less affected by the paramagnetic radical; 2) the introduction of a pulsed high power microwave source, an extended interaction klystron multiplier (EIK) for pumping with peak power up to 100 Watt. The reported experiments are well performed. The authors demonstrate that the DNP mechanism is a solid-state effect and the enhancements depend on the duty cycle (or average power) from the source. They also show a narrowing of the NMR line as compared to the widespread nitroxide radical.

My main criticism concerns the overall claim and the way the manuscript is written in some parts. The method is demonstrated on a single molecule, particularly on a methyl group that is a special case due to its fast motion/rotation. In my opinion, this is too limited to claim that the method is suited to hyperpolarize protons on small molecules, with perspective applications in metabolomics and ligand binding studies. The manuscript would strongly benefit from a few more examples on small molecules.

As detailed above we now include a range of other molecules and proton sites in the manuscript.

It should be considered that there exists a whelm of literature on d-DNP using water protons and how this can be used to polarize proteins. The citations here could be expanded (see recent papers by Frydman, Bodenhausen, Kurzbach, etc). Also, citations on state-of-the art liquid-state NMR hyperpolarization, including Overhauser and solid-effect DNP as well as PHIP in liquids, are missed.

We now cite the hyperpolarization techniques mentioned by the referee, as well as photo-CIDNP. We also refer specifically to hyperpolarized water.

Moreover, the manuscript contains statements which are not well-explained. Examples are listed below. In my view, the manuscript is not suited to Nature Communications in the present form. It might become convincing if more examples and detailed comparison with current state-of-the art in liquid-state hyperpolarization methods are added.

Specific points:

1) Introduction: 'A broadly applicable technique for liquid-state NMR is d-DNP…' I'm not sure if this statement is correct and the technique is general. There is still no general method for liquids. The authors should cite here also other approaches like solid effect in liquids (Ref. 17 is cited at the wrong place), recent progress in Overhauser DNP at high fields as well as PHIP (see above).

We have revised the sentence to read "Dissolution-dynamic nuclear polarization (d-DNP) is an indirect hyperpolarization technique for liquid-state NMR with the potential to achieve near unity spin polarization []." The strengths and weaknesses of D-DNP are detailed in the ensuing paragraph. Other hyperpolarization techniques are now referenced earlier in the introduction.

2) Introduction, page 2: why only thermal mixing is discussed and not cross-effect with biradicals ? Please explain the difference for the general reader.

A sentence explaining the relation between thermal mixing and the cross effect has been added, together with relevant references.

3) C: DNP build-ups. 'The curves represent the polarization growth associated with DNP as background..' This statement is unclear: what is the DNP as background ?

The background and the thermal equilibrium signal were subtracted. The sentence has been rewritten for improved clarity.

4) Same chapter: 'A threefold reduction in OX063 concentration…leads to a steady-state magnetization that is 3.5 larger than that obtained with 15 mM..'. This sentence seems wrong. From Figure 2, I read the opposite, ie the polarization of the 15 mM sample (green curve) is three time larger the one of the 5 mM sample (purple). Please clarify.

This error has been corrected.

5) Discussion, first paragraph. Please give numbers for concentration sensitivity instead of dilution factors. The latter cannot be easily translated in sensitivity.

After careful consideration, we have decided to put the emphasis on polarization levels rather than dilution factors or concentration sensitivities. We have added a paragraph and reference to the discussion explaining the effect of dilution (which may range from irrelevant to highly critical depending on the application). We also add a reference to an earlier study in which the influence of the NMR probe on final concentration sensitivity is discussed in greater detail.

While the concentration sensitivity of our method is often important, there are many anticipated applications in particular in metabolomics in which the available sample is precious and low dilution is important, in the sense that one wants to use the amount of liquid required to fill the NMR tube, but not excessively more. An analysis of the effect of dilution is given in the supplementary information.

6) Discussion, third paragraph. Why does a shorter T1 of the trityl radical improves the DNP process ? Why do rare earth ions only affect electron T1 and not nuclear T1 ? Please expand on this.

We now explain how a shorter T1 may benefit the DNP process. In general, the referee is right that the nuclear T1 may be affected adversely, however at least in conventional d-DNP at 3.35 T the benefits have outweighed the additional losses. We have added a corresponding comment.

7) Discussion, last paragraph: 'the high resolution provided by nitroxide radicals directly benefits applications of the methodology presented here in metabolomics..' The high resolution should refer to the OX063 radical instead of nitroxide. Moreover, the high resolution is demonstrated only on a methyl group, which is a special case as fast rotating. This statement is speculative.

The sentence was corrected. Since our expanded dataset also demonstrates improved resolution on aromatic and aliphatic protons we suggest to retain the statement.

8) Please expand the discussion and comparison to other methods for hyperpolarization in liquids.

Since we now discuss other hyperpolarization methods in greater detail in the introduction, we have chosen not to reiterate these techniques in the discussion. A comparison to other DNP methods applied to the same target molecules and solvent is given now in the supplementary information.

Reviewer #3 (Remarks to the Author):

The article uses a version of dissolution DNP to polarize samples via 1H NMR rather than 13C NMR. The article is very well written and the experiments are complete and well documented. The rationale for using 1H rather than 13C is sensitivity and the shorter polarization periods which are well known. In this sense the paper is not breaking new ground. However, with some rare exceptions the dDNP community has be religiously focuses on proceeding with 13C polarization. The "bullet" polarizers developed by Meier facilitate using 1H.

I would favor publishing the article without modifications as it does demonstrate the way that dDNP experiments should be performed.

We thank the referee for his support.
